# Comparison of Ocular Biometry Profiles in Urban and Rural Cataract Candidates in Eastern China

**DOI:** 10.1155/2020/2863698

**Published:** 2020-12-15

**Authors:** Hui Liu, Zequan Xu, Zhuyun Qian, Jianheng Liang, Kunqiao Wei, Lipiya Zu, Xu Chen

**Affiliations:** ^1^Aier School of Ophthalmology, Central South University, Changsha, Hunan Province, China; ^2^Department of Ophthalmology, Frist Medical Center, Chinese People's Liberation Army General Hospital (PLAGH), Fuxing Road 28, Beijing 100039, China; ^3^Department of Ophthalmology, Shanghai Aier Eye Hospital, Shanghai, China; ^4^Department of Ophthalmology, Wulumuqi Aier Adiya Eye Hospital, Wulumuqi, China

## Abstract

**Purpose:**

To compare ocular biometric parameters between urban and rural cataract patients in Shanghai, China.

**Methods:**

A study of ocular biometry records from urban and rural hospitals was performed for cataract patients at least 50 years of age. The ocular biometrical parameters, which were measured with partial coherence laser interferometry (IOL Master, Zeiss), were axial length (AL), anterior chamber depth (ACD), radius of corneal curvature (*K*, including steep/flat/average *K*), astigmatism, and axis. Only the right eye record of each patient was analysed.

**Results:**

Ocular biometric data included 2839 urban patients (73.15 ± 9.54 years) and 2646 rural patients (73.64 ± 7.32 years). Mean AL, ACD, and *K* were 24.35 ± 2.34 mm, 3.14 ± 0.58 mm, and 44.38 ± 1.52 D, respectively, in urban patients and 23.58 ± 1.70 mm, 3.08 ± 0.57 mm, and 44.53 ± 1.50 D, respectively, in rural patients. The urban subjects had significantly longer axial lengths (in both univariate and multivariate linear regression analyses) and deeper ACDs (in univariate analysis) than rural subjects (*p* < 0.01). There was no significant difference in steep *K*, flat *K*, and average *K* between the two groups. With-the-rule (WTR) corneal astigmatism was found in 1787 eyes (32.58%), against-the-rule (ATR) corneal astigmatism was found in 2727 eyes (49.72%), and oblique corneal astigmatism was found in 971 eyes (17.70%).

**Conclusions:**

We report biometry and astigmatism data in a large cohort of urban and rural adult subjects for the first time. In our study, a short AL, shallow ACD, and axis turned in an ATR direction had higher prevalence rates in the rural subjects. This profile of ocular biometric data and corneal astigmatism will be helpful in planning for intraocular lens (IOL) power calculations and astigmatism correction in subjects in different locations.

## 1. Background

The expectations of both surgeons and patients are high for accurate refractive results after the phacoemulsification and intraocular lenses (IOLs) implantation surgery. One important key for these successful outcomes is obtaining accurate ocular biometric measurements preoperatively [1]. The basic ocular biometric characteristics include axial length (AL), anterior chamber depth (ACD), keratometric power (*K*), and corneal astigmatism (CA) [1]. The modern IOL power formula, which is based on the Gaussian model, predicts the effective lens position (ELP) by using these biometric parameters. With modern biometry and the new-generation IOL power formulas, the refractive outcomes in approximately 87% of patients are within ± 1.0 diopter (D) of the intended target [2].

The specific AL, *K*, and ACD averages according to the gender and race of patients in different areas may influence the accuracy of the IOL power calculation even when using the same formula. Studies concerning the distribution of ocular biometrics in different populations from Asian countries (Mongolia, Myanmar, Singapore, and China) have been published [3–6]. In China, the comparable biometric data of interethnic variability in a large population-based study are still lacking.

The purpose of our study was to assess the distribution of AL and other ocular biometric parameters, as measured using the partial coherence laser interferometry (IOLMaster, Carl Zeiss Meditec, Germany), in the urban and rural populations of the municipality of Shanghai in eastern China.

## 2. Patients and Methods

### 2.1. Subjects

This study was approved by the Ethics Committee of the Shanghai Aier Hospital (Shanghai, China) and followed the tenets of the Declaration of Helsinki. Consecutive cataract patients scheduled for phacoemulsification and foldable IOL implantation were recruited in the urban location of Shanghai Aier Eye Hospital and in the rural location of Shanghai Jinshan Aier Eye Hospital between January 2018 and December 2018. Only patients aged over 50 years were included. Exclusion criteria included a history of ocular surgery, such as refractive surgery, corneal diseases, ocular inflammation, and trauma; patients from other areas of China were also excluded. Routine eye slit-lamp and biometry examinations were performed before surgery. The procedures were fully explained to each patient, and they provided written informed consent.

### 2.2. Biometry Examination

Ocular axial length (AL), anterior chamber depth (ACD), radius of corneal curvature (*K*, including steep/flat/average *K*), astigmatism, and axis of the right eye were measured with the IOL Master 500 (Carl Zeiss Meditec, Germany, software version 5.4) in undilated pupils. The mean value of 5 measurements was used for each parameter. All patients were tested by the same experienced examiner. Keratometry, including flat keratometry (*K*1) and steep keratometry (*K*2), was recorded. The *K* value was calculated as the mean of *K*1 and *K*2.

### 2.3. Statistical Analysis

All data were recorded in Microsoft Excel spreadsheets. Statistical analysis was performed using SPSS PASW Statistics version 18.0 software (IBM Corporation, Armonk, NY, USA). Distributions of normality of the ocular biometric parameters were checked with the Kolmogorov–Smirnov (K-S) test and were considered significantly different from normal when the *p* value was less than 0.05. Differences between groups were compared using the *t*-test or analysis of variance (ANOVA) for normally distributed variables and the Mann–Whitney *U* test for nonnormally distributed variables. One-way analysis of variance and the Kruskal–Wallis test were, respectively, applied for the comparison of variance for normally and nonnormally distributed data among the different age groups. Comparison between any two age groups of *K* value was carried out by the SNK multiple comparison test (for data with homogeneity of variance) or Tamhane multiple comparison test (for data with heterogeneity of variance). Possible correlations between biometric parameters were assessed using Spearman's correlation coefficient. Associations between ocular and systemic factors with AL, ACD, and *K* were assessed using multivariate linear regression models. A *p* value less than 0.05 was considered statistically significant.

## 3. Results

### 3.1. Demographics of the Study Population

We enrolled 7217 eyes of 7217 patients (3355 eyes from urban areas and 3862 eyes from rural areas); 1732 cases subsequently had to be excluded because of the impossibility to achieve a correct AL measurement (dense cataract). Thus, the final analysis was performed on 5485 right eyes of 5485 participants (2839 eyes from urban areas and 2646 eyes from rural areas).  Table 1 shows the patient demographics of all participants and provides a comparison of the rural and urban populations. The population was stratified by age: 50–59 years (7.06% of the total population, mean age 55.57 ± 2.80 years); 60–69 years (22.92%, 65.07 ± 2.67 years); 70–79 years (44.23%, 74.40 ± 2.84 years); and 80+ years (25.80%, 83.53 ± 3.04 years). There were no significant differences between the age groups.

### 3.2. Distribution of Ocular Axial Length Characteristics

**Figure 1:**
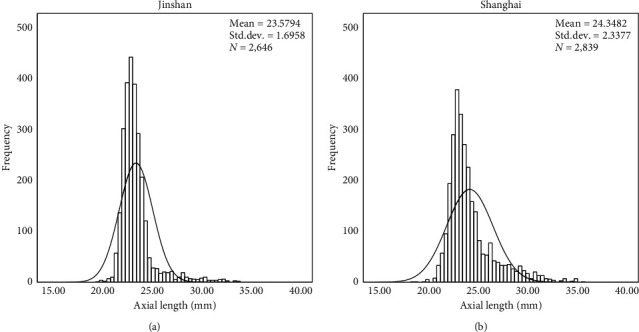
Distribution of axial length in the total Shanghai population.

Figure 1 shows the distribution of ocular axial length in the whole population. The AL distribution (mean: 23.98 mm; median: 23.41 mm; range: 18.45–35.64 mm) was skewed toward the right (2.09), peaked with a kurtosis value of 5.31, and had a significant Kolmogorov–Smirnov test for deviation from normality (*p* < 0.01).

In the urban group, the mean AL was 24.35 mm (median: 23.67 mm; range: 18.45–35.64 mm), and in the rural group, the mean AL was 23.58 mm (median: 23.23 mm; range: 19.87–33.92 mm), showing a statistically significant location-related difference (*p* < 0.001, Mann–Whitney *U* test).

In the urban group, the mean AL in women was 24.18 ± 2.48 mm (95% CI: 24.06–24.30 mm), and the mean AL in men was 24.61 ± 2.06 mm (95% CI: 24.49–24.73 mm), showing a statistically significant gender-related difference (*p* < 0.001). In the rural group, the mean AL in women was 23.40 ± 1.69 mm (95% CI: 23.32–23.49 mm), and the mean AL in men was 23.86 ± 1.66 mm (95% CI: 23.75–23.96 mm), showing a statistically significant gender-related difference (*p* < 0.001). Mean AL was significantly longer in men than in women in both groups (Table 2).

### 3.3. Distribution of Ocular ACD Characteristics

Table 2 shows the ACD distribution (mean: 3.11 mm, 95% CI, range 3.10–3.13 mm) which was not normal distribution in either group (urban: 3.14 ± 0.58 mm; rural: 3.08 ± 0.57 mm) or the whole population (Kolmogorov–Smirnov test, *p* < 0.001).

**Figure 2:**
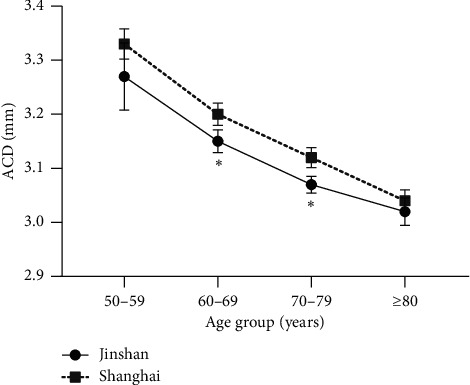
Distribution of ACD in Shanghai and Jinshan populations in all age groups.

In the urban group, a decrease of ACD value was associated with increased age in men, in women, and in the combined group. A similar decrease in ACD value within a decade of age in different groups was also shown in the rural population. In the 60- to 69-year group and the 70- to 79-year group, there were significant differences in ACD between the different location populations. In the 50- to 59-year group and the 80+-year group, the differences were not significant between the two locations (Figure 2).

### 3.4. Distribution of Corneal *K* Characteristics

The mean *K* of the overall population was 44.45 diopters (D) (95% CI, 44.41–44.49 D), and the distribution was right (positively) skewed (0.92) with a kurtosis value of 0.263 (K-S test, *p* < 0.001). As shown in Table 2, the mean *K* reading of the rural population (44.53 ± 1.50 D) was significantly different from that of the urban population (44.38 ± 1.52 D, *p* < 0.001). The mean *K* reading was also significantly different between the male and female populations in the urban area, rural area, and combination groups.

In Table 3, the mean *K* reading was shown across age and location groups. There are significant differences in the *K* reading between the different age groups in the urban area population, while there are no significant differences in the *K* reading between the different age groups in the rural area population.

### 3.5. Distribution of Corneal Astigmatism (CA) Characteristics

The CA distribution (mean, 1.07 ± 0.72 D) was skewed towards the positive (1.559) and peaked with a kurtosis value of 3.652 (K-S test, *p* < 0.001).

**Figure 3:**
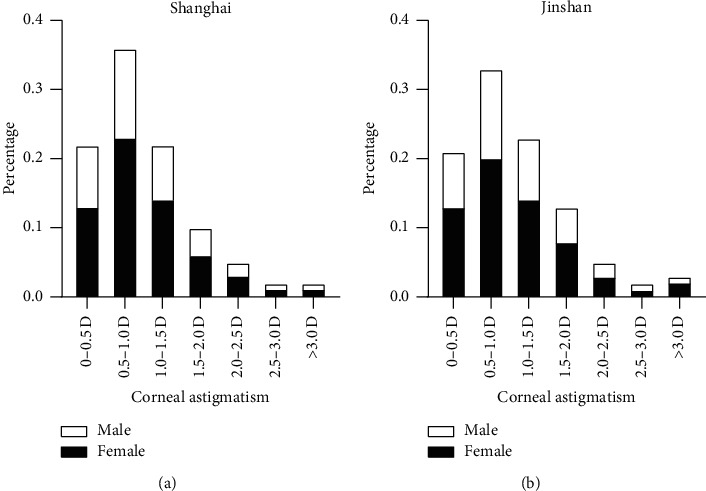
Distribution of corneal astigmatism in the total Shanghai population.

The histograms of the frequency distribution of corneal astigmatism for all location groups are shown in Figure 3. Corneal astigmatism of 0.51–1.00 D was the most common range of values in both urban and rural areas (35.7% and 33.0%, respectively). In urban areas, the next most common ranges were 0.00–0.50 D (22.4%), 1.01–1.50 D (22.1%), and 1.51–2.00 D (13.0%), while for rural areas, the next most common ranges were 1.01–1.50 D (23.1%), 0.0–0.50 D (21.1%), and 1.51–2.00 D (13.0%).

**Figure 4:**
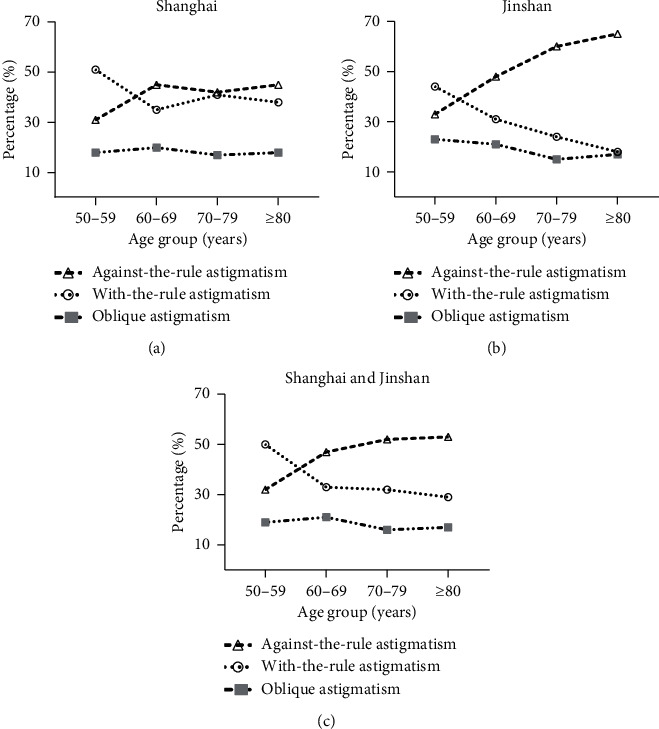
Percentages of with-the-rule, against-the-rule, and oblique corneal astigmatisms in the 4 groups.

With-the-rule (WTR, the steep meridian of the cornea being within 90 ± 30 degrees) corneal astigmatism was found in 1787 eyes (32.58%), against-the-rule (ATR, the steep meridian of the cornea being within 180 ± 30 degrees) corneal astigmatism was found in 2727 eyes (49.72%), and oblique (neither WTR nor ATR) corneal astigmatism was found in 971 eyes (17.70%). The prevalence of WTR corneal astigmatism decreased with increasing age, while the prevalence of ATR corneal astigmatism rose. The trend of ART/WTR changing across age groups was more obvious in the rural population than in the urban (Figure 4).

### 3.6. Correlations

There was a statistically significant negative correlation between AL, ACD, and patient age (AL: −0.060, *p* < 0.01; ACD: −0.158, *p* < 0.01). However, CA was positively correlated with age (0.184, *p* < 0.01). AL correlated positively with ACD (0.496, *p* < 0.01) and with CA (0.052, *p* < 0.01), but negatively with *K* (−0.412, *p* < 0.01). *K* correlated negatively with ACD (−0.066, *p* < 0.01) and positively with CA (0.056, *p* < 0.01). A higher astigmatism was associated with deeper ACD (0.042, *p* < 0.01, Spearman test).

Tables 4–7 show the associations of AL, ACD, *K*, and CA with ocular and systemic factors. Multivariate linear regression analysis showed that younger, male sex, steeper corneal curvature, and longer axial length were independently associated with deeper ACD (all *p* < 0.05). The ACD in rural was 0.029 mm deeper than that in urban (*p* < 0.05), and AL in urban was 0.654 mm longer than that in rural (*p* < 0.001), after adjusted for other factors included in the regression model. There is no significant difference in *K* readings between the different regions in multivariate linear regression analysis (*p*=0.494). But the significant difference was demonstrated in other ocular parameters such as AL, ACD, and the age/gender (all *p* < 0.05). The significant difference in CA was showed in age, location, and ocular parameters in multivariate linear regression analysis (all *p* < 0.05).

## 4. Discussion

Our study evaluated the ocular biometric data characteristics in Shanghai urban and rural populations by using a partial coherence laser interferometry (PCI). PCI is widely used in clinical work due to its highly accurate and reproducible measurements. The accuracy of the biometric data leads to accurate calculations of IOL power. Previous studies have investigated the characteristics of biometric data of different race populations, such as southern Chinese [7], Latin American [8], Malay [6], Indian [9], Western populations [10], and American [11]. To our knowledge, this is the first biometry study that focuses on urban and rural cataract patients in China.

We demonstrated that the biometry parameters, such as AL, ACD, *K*, and CA, were distributed nonnormally in the general investigated population (all participants), in the different location populations (urban versus rural), and in the gender populations (male versus female), respectively.

The AL data in our study was positively skewed and showed significant kurtosis, as reported in the Reykjavík eye study [12] and the studies by Fotedar [13] and Chen [14]. In the combination/general population, the mean AL was 23.98 ± 2.09 mm, which was similar to the results of the study in southern China by Cui et al. (24.07 ± 2.14 mm) [7]. But the mean AL in urban areas was about 0.8 mm longer than that in rural areas (24.35 ± 2.34 mm versus 23.58 ± 1.70 mm, respectively). Mean AL in Shanghai was similar to Yu's study (24.38 ± 2.47 mm) from central China [15] and Huang's study (24.32 ± 2.42 mm) from western China [16], but was clearly longer than other AL studies using PCI [6, 9, 10], which investigated other ethnicities, such as European (23.43 ± 1.51 mm) [10], Latin American (23.8 mm) [8], Malay (23.55 mm) [6], and Mongolian (23.13 ± 1.15 mm) [4].

Yu [15] and Huang [16] had investigated the inhabitants in Wuhan and Chengdu urban areas, respectively, which are similar in latitude to Shanghai. All of these cities belong to the reaches of the Yangtze River. For other Chinese inhabitants in urban southern China, on the reaches of Zhu River, and other Asian countries, mean AL is shorter than for those living in Shanghai.

According to many previous studies [4, 17], axial length was strongly correlated with the refraction status. Because our study was based on cataract surgery candidates who had not been evaluated the refractive error correctly, we did not collect the refractive data. No advanced analysis about the relationship between refractive error and biometry was performed. That is a major weakness of our study. Some researchers reported previously that educational status was the strongest determinant of AL, and AL was also significantly associated with body height [18]. In addition, Liu reported that urban children have higher height than rural children, considering that nutritional status is one of the reasons for this difference [19]. A recent study reported similar results that children from urban regions had significantly longer eyes than rural children [20]. Though these parameters are not available in our results, we speculate AL should be related to multiple causes such as refractive status, body shape, education, and environment between the urban population and the rural population.

As for the gender factor, we found that men had a longer AL than women, which is consistent with findings from previous studies [7, 13, 16]. These disparities reflect the different physical conditions between the sexes.

We found that the ACD was deeper in a younger, male, urban population than it was in an older, female, rural population. The first two findings were similar to other studies [4, 13, 17]. Men had deeper anterior chamber depth than women, which was statistically significant in both univariate and multivariate analyses, and it was attributed to differences of male and female anatomy, particularly height. The depth of the ACD often decreased with an increase in age-related lens thickness, which can be observed in any gender or race [4, 13, 17]. In comparison with European (3.11 ± 0.43 mm) [10], Austrian (3.10 mm) [13], and Latin American studies (3.41 ± 0.35 mm) [8], the mean ACD depth is shallower in Shanghai and Jinshan. Previous studies found that elderly Asian women were more likely to develop acute angle-closure glaucoma due to factors of sex and race, which is similar to the results of Chinese population studies from Cui (3.01 ± 0.57 mm) [7] and Chen (3.03 mm) [14]. However, other investigators, such as Huang (3.08 ± 0.47 mm) [16] and Yu (3.15 ± 0.48 mm) [15], found a deeper ACD than we did.

As for the differences in ACD between the urban and rural populations, we speculate that it may be caused by two reasons. First, it may be caused by differences of myopia in the prevalence between the two groups. Xu et al. had demonstrated that myopic refractive error was significantly associated with younger age and an urban region [21]. In He's study, refractive error was strongly correlated with axial length and anterior chamber depth by using the multivariate models [22]. We are not sure that myopia prevalence is the source of the difference because the refractive data were not collected in our study. We found that the ACD in rural was deeper than that in urban after adjusted for other factors (such as ages, gender, and axial length) included in the regression model, and it is inconsistent with the results of univariate analysis. Therefore, we consider the differences in ACD may be strongly related to the AL distribution in the two populations. Second, the increase in lens thickness was accompanied by a decrease in anterior chamber depth [23]. However, the measurement of lens thickness is not possible in the IOL Master 500. Thus, the difference between lens thicknesses could not be obtained.

In our study, we found that 44.18% of the overall population had 1.0 D or more corneal astigmatism, which is higher than other Chinese population results. Chen [24] reported that 41.3% of the eyes studied had presented a corneal astigmatism equal to or higher than 1.0 D, while Cui and Yu reported 43.9% and 43.5%, respectively [7, 15]. Two other Chinese studies showed a higher percentage of eyes with over 1.0 D astigmatism than our study (Guan 45.46% and Yuan 47.27%) [25, 26]. We speculate that the difference between these studies is caused by two factors: first, these studies focused on a larger age range than our study, and second, these studies recruited many cases that included both eyes, which may increase the statistical power of a test, thereby increasing the likelihood of detecting true significant effects (Table 8). In multivariate linear regression analysis, both *K* reading and CA showed a significant difference in ocular parameters and age. These differences should be investigated in the further study.

In our study, we found that the proportion of eyes in urban areas that had a corneal astigmatism of 1.0 D or greater was 41.9%, which was less than the proportion in rural areas (45.9%). We also found the astigmatism axis turned to the ATR direction with age. This trend of ATR increasing as subjects grow older has been proven by many previous investigations. However, a higher percentage of ATR astigmatism was found in the rural population than in the urban population. In the population with 1.0 D or more astigmatism in urban areas, 42.25% of patients had WTR and 46.16% had ATR. In rural areas in the same population, 23.14% of patients had WTR and 67.05% had ATR. Previous studies have demonstrated a higher percentage of ATR astigmatism in an Asian population (49.7% in our study, 53.2% in Cui's study and 62.2% in a Thailand population) [7, 27] than in Western populations. There are many factors that influence whether the astigmatism axis direction turns with-the-rule or against-the-rule, such as ethnicity, anatomy, eyelid morphology, and the effects of intraocular pressure on the curvature of the cornea. The reasons that have led to the difference in the prevalence of ATR in various locations still need to be investigated further. In our study, the differences of astigmatism between the two groups may be caused by two factors: firstly, although there was no statistical difference in age between the two groups, the overall age of patients in the rural was higher than in the urban areas, which may lead to an increase of ATR; secondly, it may be related to the prevalence of myopia between the two groups. Cataract surgeons should consider using more toric IOLs in rural populations than in urban populations.

Our study has some limitations. First, we cannot exclude selection bias because the study was clinic-based and may not be representative of the entire population. In addition, in the 50- to 59-year age group, the sample size of rural is much smaller than that of urban, which may not represent the general population of this age group. Second, the relationship between biometric features and refraction was not evaluated due to the cloudy crystalline lens of the cataract patients. There were also missing measurements, such as the white-to-white, central corneal thickness, lens thickness, and vitreous chamber depth. Third, IOL Master measures only 6 points on the cornea and is not as exact for evaluating corneal structure as other methods such as corneal topography. Finally, anthropometric parameters (such as body height and weight), social status, education, and occupation were not recorded in our study.

## 5. Conclusion

We report biometry and astigmatism data in a large cohort of urban and rural adult subjects for the first time. In our study, the rural subjects were more likely to have a short AL, shallow ACD, and an axis turned in an ATR direction. This profile of ocular biometric data and corneal astigmatism will be helpful in planning IOL power calculations and astigmatism correction in patients who live in different locations.

## Figures and Tables

**Table 1:** Demographic features of the present study population.

Age group (years)/location		Shanghai (urban)	Jinshan (rural)	Total
All	Male/female	1095/1744	1030/1616	2125/3360
	Patients (eyes)	2839	2646	5485
	Age	73.15 ± 9.54	73.64 ± 7.32	73.29 ± 8.56

50–59	Eyes	330	57	387 (7.06%)
	Age	55.58 ± 2.83	55.51 ± 2.63	55.57 ± 2.80

60–69	Eyes	601	656	1257 (22.92%)
	Age	64.95 ± 2.71	65.17 ± 2.62	65.07 ± 2.67

70–79	Eyes	1072	1354	2426 (44.23%)
	Age	74.48 ± 2.88	74.34 ± 2.81	74.40 ± 2.84

>=80	Eyes	836	579	1415 (25.80%)
	Age	83.64 ± 3.17	83.38 ± 2.84	83.53 ± 3.04

**Table 2:** Sex distribution of ocular biometric parameters.

Location/gender		Mean ± SD					
		AL (mm)	ACD (mm)	Astigmatism (D)	*K* (D)		
					*K* mean	*K*1	*K*2
Jinshan and Shanghai	All (5485)	23.98 ± 2.09	3.11 ± 2.09	1.07 ± 0.72	44.45 ± 1.51	43.92 ± 1.54	44.98 ± 1.58
	M (3360)	24.25 ± 1.91	3.20 ± 0.58	1.06 ± 0.70	44.00 ± 1.49	43.47 ± 1.51	44.53 ± 1.55
	F (2125)	23.81 ± 2.17	3.05 ± 0.57	1.07 ± 0.73	44.74 ± 1.16	44.20 ± 1.49	45.27 ± 1.53
	*p* value (sex)	<0.001	<0.001	0.791	<0.001	<0.001	<0.001

Jinshan (rural)	All (2676)	23.58 ± 1.70	3.08 ± 0.57	1.09 ± 0.73	44.53 ± 1.50	43.98 ± 1.54	45.07 ± 1.55
	M (1030)	23.86 ± 1.66	3.17 ± 0.57	1.08 ± 0.68	44.04 ± 1.42	43.50 ± 1.47	44.58 ± 1.46
	F (1616)	23.40 ± 1.69	3.03 ± 0.57	1.10 ± 0.76	44.84 ± 1.46	44.29 ± 1.50	45.39 ± 1.53
	*p* value (sex)	<0.001	<0.001	0.712	<0.001	<0.001	<0.001

Shanghai (urban)	All (2839)	24.35 ± 2.34	3.14 ± 0.58	1.04 ± 0.70	44.38 ± 1.52	43.86 ± 1.53	44.90 ± 1.60
	M (1095)	24.61 ± 2.06	3.24 ± 0.58	1.04 ± 0.72	43.96 ± 1.54	43.44 ± 1.54	44.29 ± 1.63
	F (1744)	24.18 ± 2.48	3.08 ± 0.57	1.05 ± 0.70	44.64 ± 1.45	44.12 ± 1.47	45.17 ± 1.52
	*p* value (sex)	<0.001	<0.001	0.422	<0.001	<0.001	<0.001

*p* value^*∗*^		<0.001	<0.001	0.010	<0.001	0.003	<0.001

ACD: anterior chamber depth; AL: axial length; *K*1: flat keratometry; *K*2: steep keratometry; D: diopter; M: male; F: female. ^*∗*^Statistical significance of difference between the rural and urban groups.

**Table 3:** Descriptive statistics of mean *K* reading in 4 age groups.

Age group (years)	Shanghai (urban)	Jinshan (rural)
	*K* (D) mean ± SD	*K* (D) mean ± SD
Group A (50–59)	44.11 ± 1.57^#+^	44.52 ± 1.75
Group B (60–69)	44.56 ± 1.50^#^^*∗*^	44.44 ± 1.55
Group C (70–79)	44.40 ± 1.57^+^	44.56 ± 1.51
Group D (>=80)	44.34 ± 1.45^*∗*^	44.54 ± 1.40
*p* value	<0.001	0.427

*K* mean: keratometry; D: diopter; SD: standard deviation; *p* value represents the difference among four age groups of Shanghai/Jinshan; ^#^*p* < 0.05 Group A vs. Group B. ^+^*p* < 0.05 Group A vs. Group C. ^*∗*^*p* < 0.05 Group B vs. Group D. Multiple comparison between each two age groups in Jinshan showed no statistical difference; multiple comparison between each other two age groups in Shanghai showed no statistical difference.

**Table 4:** Associations of AL with systemic and ocular factors.

		Multivariate model		
		*β*	95% CI	*p* value
Age (years)		−0.031	−0.036 to −0.025	<0.001
Gender	Male	Reference		
	Female	−0.106	−0.208 to −0.003	0.043
Keratometric power (D)		−0.254	−0.287 to −0.221	<0.001
Anterior chamber depth (mm)		1.312	1.227 to 1.397	<0.001
Astigmatism (D)		0.312	0.244 to 0.381	<0.001
Location	Shanghai (urban)	Reference		
	Jinshan (rural)	−0.654	−0.750 to −0.558	<0.001

**Table 5:** Associations of ACD with systemic and ocular factors.

		Multivariate model		
		*β*	95% CI	*p* value
Age (years)		−0.005	−0.007 to −0.004	<0.001
Gender	Male	Reference		
	Female	−0.116	−0.146 to −0.087	<0.001
Axial length (mm)		0.109	0.102 to 0.116	<0.001
Keratometric power (D)		0.015	0.006 to 0.025	0.002
Astigmatism (D)		0.031	0.011 to 0.051	0.002
Location	Shanghai (urban)	Reference		
	Jinshan (rural)	0.029	0.001 to 0.057	0.045

**Table 6:** Associations of *K* with systemic and ocular factors.

		Multivariate model		
		*β*	95% CI	*p* value
Age (years)		−0.004	0.009 to 0.000	0.069
Gender	Male	Reference		
	Female	0.674	0.595 to 0.754	<0.001
Axial length (mm)		−0.160	−0.180 to −0.139	<0.001
Anterior chamber depth (mm)		0.115	0.043 to 0.188	0.002
Astigmatism (D)		0.168	0.113 to 0.222	<0.001
Location	Shanghai (urban)	Reference		
	Jinshan (rural)	0.027	−0.050 to 0.105	0.494

**Table 7:** Associations of CA with systemic and ocular factors.

		Multivariate model		
		*β*	95% CI	*p* value
Age (years)		0.017	0.014 to 0.019	<0.001
Gender	Male	Reference		
	Female	0.034	−0.006 to 0.073	0.096
Axial length (mm)		0.047	0.036 to 0.057	<0.001
Anterior chamber depth (mm)		0.056	0.020 to 0.091	0.002
Keratometric power (D)		0.040	0.027 to 0.053	<0.001
Location	Shanghai (urban)	Reference		
	Jinshan (rural)	0.067	0.029 to 0.105	0.001

CI: confidence interval; AL: axial length; ACD: anterior chamber depth; *K* mean: keratometric power; CA: cornea astigmatism.

**Table 8:** Patient ocular biometric data compared with other published studies.

	Location	Eyes	Age (years)		Keratometry (D)			AL (mm)		ACD (mm)	
			Mean ± SD	Range	*K*1-mean ± SD	*K*2-mean ± SD	*K*-mean ± SD	Male	Female	Male	Female
Yu et al. [15]	Central China	3209	70.51 ± 9.81	32.95	43.75 ± 1.59	44.84 ± 1.65	44.29 ± 1.58	24.38 ± 2.47		3.15 ± 0.48	
Lim et al. [6]	Southern China	6750	70.40 ± 10.50	40.101	43.57 ± 1.69	44.69 ± 1.69	44.13 ± 1.63	24.28 ± 2.08	23.90 ± 2.18	3.08 ± 0.59	2.96 ± 0.55
Guan et al. [25]	Eastern China	1430	72.27 ± 11.59	16.98	43.57 ± 1.56	44.64 ± 1.65	NR	NR		NR	
Yuan et al. [26]	Northern China	12449	69.80 ± 11.15	30.97	43.93 ± 1.67	45.08 ± 1.73	NR	NR		NR	
Huang et al. [16]	Western China	6933	NR	50.98	NR	NR	44.23 ± 1.66	24.79 ± 2.48	23.88 ± 2.27	3.16 ± 0.47	3.01 ± 0.47
Pan et al. [9]	Singapore	3400	NR	40.83	43.95	44.7	NR	23.68 ± 1.06	23.23 ± 1.10	3.19 ± 0.36	3.10 ± 0.35
Fotedar et al. [13]	Australian	1321	NR	≥59	43.38	NR	NR	23.75	23.20	3.16	3.06
Warrier et al. [3]	Myanmar	1498	NR	≥40	—	NR	—	23.12	22.54	2.86	2.29
Cui et al. [7]	Los Angeles	5588	NR	≥40	—	NR	—	23.65 ± 0.94	23.18 ± 1.02	3.48 ± 0.34	3.36 ± 0.34
Lee et al. [11]	Beaver Dam	2375	71.90 ± 9.10	58.100	—	NR	—	23.92 ± 1.10	23.51 ± 1.17	3.14 ± 0.38	3.09 ± 0.36

D: diopter; *K*1: flat keratometry; *K*2: steep keratometry; *K* mean: keratometry; SD: standard deviation; AL: axial length; ACD: anterior chamber depth; NR: not reported.

## Data Availability

The data used to support the findings of this study have not been made available because the data also forms part of an ongoing study.
